# Revision total knee replacement case-mix at a major revision centre

**DOI:** 10.1186/s40634-022-00462-2

**Published:** 2022-04-14

**Authors:** Shiraz A. Sabah, Lennart von Fritsch, Tanvir Khan, Alexander D. Shearman, Raja Bhaskara Rajasekaran, Oxford Revision Arthroplasty Group, Adrian Taylor, Ben Kendrick, William F. M. Jackson, Nicholas Bottomley, Antony J. R. Palmer, Christopher L. M. H. Gibbons, David W. Murray, David J. Beard, Andrew J. Price, Abtin Alvand

**Affiliations:** 1 Nuffield Department of Orthopaedics, Rheumatology and Musculoskeletal Sciences, University of Oxford, Oxford, England; 2 Nuffield Orthopaedic Centre, Oxford, England

## Introduction

Revision total knee replacement (rTKR) encompasses a diverse set of surgical procedures performed for a wide range of indications. These range from relatively minor, low-risk interventions to complex, expensive, high-risk surgeries [1, 2]. Accordingly, the clinical outcomes following surgery are highly variable and often inferior to those observed after primary TKR [3]. This combination of variable (and sometimes poor) outcomes, high costs and the morbidity of these procedures has placed a spotlight on the potential for improvement in the rTKR pathway. One solution that has received significant attention is the proposed introduction of clinical networks [4, 5]. This refers to a regional system where rTKR is delivered by a smaller number of surgeons, each working within designated units. The guiding principles underpinning this model of care include the triage of complex work to specialist centres and the avoidance of low-volume practice [4, 5]. This concept for healthcare delivery is familiar from many other areas of the NHS, including cardiovascular, cancer and trauma networks [6, 7].

The planning for a rTKR network has included characterisation of operative caseloads for surgeons and units currently performing rTKR [8]. There is some evidence to suggest that higher-volume units may achieve better outcomes following surgery [9]. However, the relationship between operating volume and surgical outcome is unlikely to be straightforward. Higher-volume centres are more likely to be presented with complex cases [10], which might be expected to have poorer outcomes. Similarly, centres with more modest caseloads and well-organised multidisciplinary teams might be able to achieve good outcomes for patients, maintain continuity of care and minimise travel for patients [11]. As such, as well as looking at minimum operating volumes, it is also important to examine individual cases themselves in more detail. A recent study from the Southwest of England [10] used the Revision Knee Complexity Classification (RKCC) [12] to examine regional rTKR practice. They found that, to some extent, a network model was already in existence in their region, with more complex cases and higher volumes of rTKR surgery being performed at their high-volume centre. However, it is not clear whether similar networks have evolved in other regions.

The aims of our study were to answer the following research questions: How complex are rTKR cases at our Major Revision Centre (MRC)? What are the current patterns of referral? Do more complex cases use greater hospital resources?

## Patients and methods

Institutional Review Board approval was obtained for this study.

### Data sources

We performed a retrospective analysis of the revision knee replacement database at the Nuffield Orthopaedic Centre, Oxford over a four-year period from 1^st^ January 2015 to 31^st^ December 2018. This database was created from revision procedures identified from theatre logbooks and the electronic theatre booking system. The number of procedures in the database was compared to the number of procedures identified in summary-level Hospital Episode Statistics Admitted Patient Care (HES APC) data at the centre over the study period to calculate the rate of case ascertainment [13]. The Office of Population Censuses and Surveys Classification of Surgical Operations and Procedures (OPCS-4) codes used by NHS Digital to produce these summary figures is provided in Appendix 1.

### Inclusion/Exclusion criteria

Revision knee replacement was defined as any procedure to add, exchange or modify an existing joint replacement. This aligns with the latest National Joint Registry definition [14]. As such, debridement, antibiotic and implant retention (DAIR) procedures with or without modular exchange, and the replacement of a further compartment of the knee (such as secondary patella resurfacing or addition of another unicompartmental knee replacement to an existing one) were classified as revision procedures. Revision knee arthroplasty procedures for malignancy were excluded.

### Patient demographics

Data were extracted on the following variables: age at revision surgery, gender, body mass index (BMI), and Charlson comorbidity index (Summary Hospital-Level Mortality Indicator [SHMI] specification [15]). Since the focus of this paper was on the activity of the surgical unit, each procedure was considered to have been performed in a unique patient for summary purposes. This meant that patients who underwent multiple rTKR over the study period (including staged procedures) contributed more than once to summary statistics.

### Indication for surgery

For each revision procedure, the indication for surgery was defined as a single, dominant diagnosis based on the Australian Orthopaedic Association National Joint Replacement Registry (AOANJRR) hierarchical system [16] (Appendix 2).

### Referral patterns

Non-urgent referrals were received via formal written communication to our department from primary or secondary care. Referrals were triaged by a consultant surgeon and then assessed in the outpatient department. Urgent referrals were received through verbal or written communication to our on-call team, and assessed in our Emergency Department or directly transferred as an inpatient to the surgical ward. We classified revision knee replacement procedures as ‘unplanned’ (for acute admissions via the Emergency Department, and inpatient transfers from other centres) or ‘elective’ (for patients undergoing planned surgery after assessment in the outpatient department).

For patients who were referred to the major revision centre over the study period, the referral source was classified as follows: primary care, primary arthroplasty unit (< 20 revisions per year), revision unit (20–70 revisions per year), and major revision centre (> 70 revisions per year). The revision volume thresholds for classification represent those proposed by the British Association for Surgeons of the Knee (BASK) Revision Knee Working Group [8]. Classification was based on publicly available 36-month practice profiles from the NJR website [14]. For each case, the number of previous joint replacement procedures on the affected knee was recorded as: first, second, third, or fourth or more.

### Technical details of revision surgery

The type of revision procedure was recorded in the same format as the NJR K2 MDS version 7 form [17]. The level of constraint of the revision construct and type of implants used were extracted for single stage and stage 2/2 procedures. The complexity of each case was rated according to the Revision Knee Complexity Classification (RKCC) after review of the electronic patient record and patient imaging (Appendix 3) [12]. This provided a three-point ordinal scale where the complexity of surgery was rated as “less complex” (R1), “complex” (R2) or “more complex/salvage” (R3) according to patient, implant and surgical factors. Each case was rated by one post-certificate for completion of training (CCT) Fellow in knee arthroplasty as well as the senior author (AA) who specialises in revision knee surgery.

### Hospital admission impact

Length of stay was recorded for each case and any usage of higher-dependency care. The relationship between length of stay and complexity as rated using the RKCC was presented visually as the cumulative probability of hospital discharge over time using the Stata package *distplot.* The strength and direction of correlation between RKCC and length of stay was measured using Spearman’s rank correlation coefficient.

### Statistical analysis

Descriptive statistics and figures were prepared using Stata (StataCorp. 2019. *Stata Statistical Software: Release 16*. College Station, TX: StataCorp LLC.) and R version 3.6.2.

## Results

**Fig. 1:**
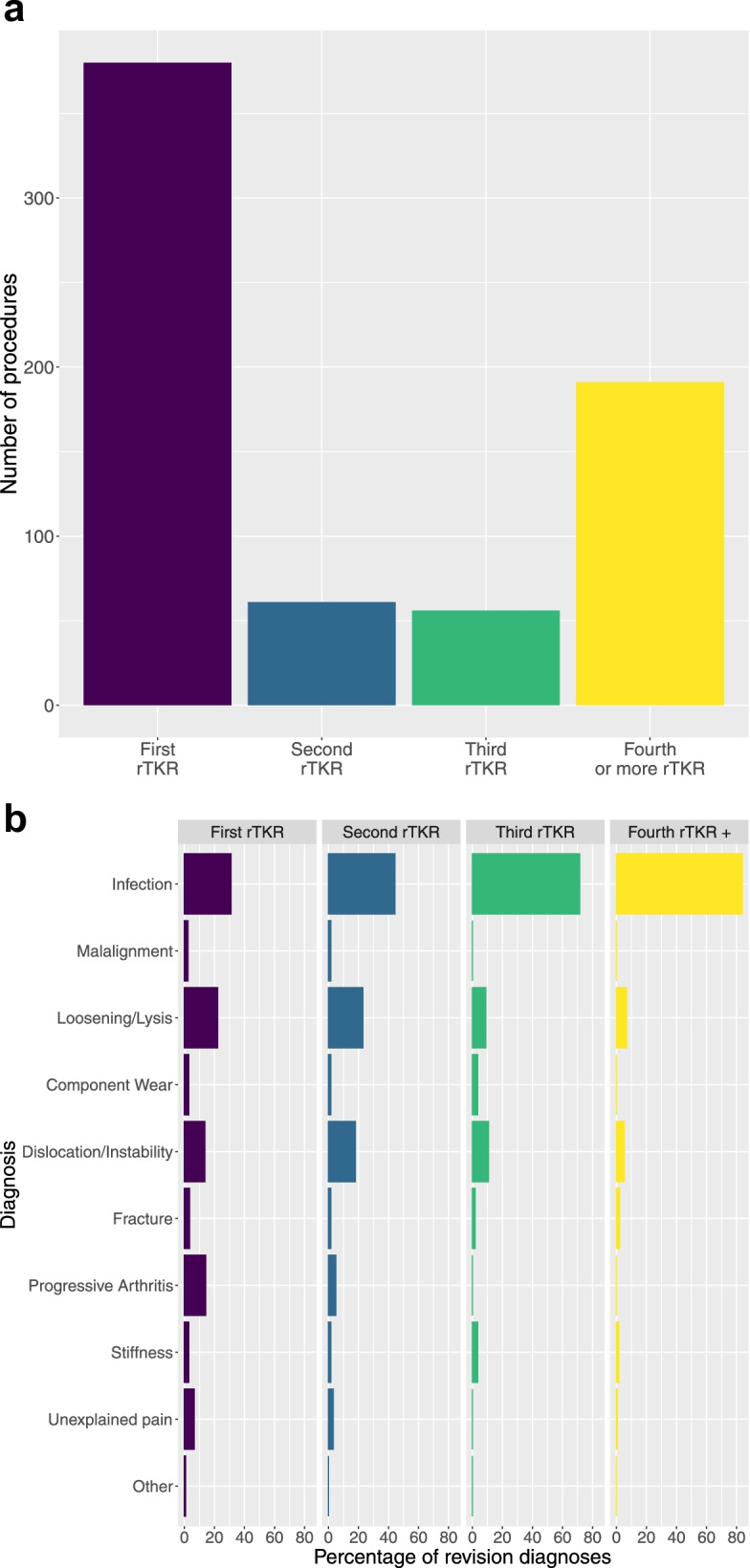
**a** Bar chart demonstrating the numbers of first revision, second revision and third or more revision TKR procedure performed in the major revision centre over the study period. **b** Tabplot’ demonstrating the indications for revision TKR. Column percentages are presented for first and subsequent revision procedures and sum vertically to 100%. Prosthetic joint infection was the most prevalent indication for revision, accounting for 83.8% of fourth or more revision procedures

Six hundred eighty-eight rTKR procedures were performed over the study period in 534 distinct patients. These comprised 380 (55.2%) first-revision procedures and 308 (44.8%) re-revision procedures (Fig. 1a).

### Case ascertainment

The local rTKR database identified 188 procedures performed in 2016/7 financial year, compared to 121 procedures reported in HES, representing case ascertainment of 155.4%. Similarly, 189 procedures were identified on the local database in 2017/8, compared to 140 procedures reported in HES (case ascertainment 135.0%).

### Patient demographics

The mean age at revision surgery was 69.5 years (standard deviation 11.8). 342 procedures (49.7%) were in female patients. 383 procedures (55.7%) were right-sided. The mean body mass index was 30.8 (sd 6.1) [data available for 670/688 procedures (97.4%)]. The median Charlson comorbidity index was 0 (IQR 0–4). Patient demographics are summarised in Table 1.

**Table 1:** Patient demographics for revision and re-revision total knee replacements at a major revision centre

	**Revision TKR procedures**
	*n* = *688*
**Gender** *(no. [%])*	Female: 342 (49.7%)
	Male: 346 (50.3%)
**Age at revision surgery**^**a**^ years *(mean [sd])*	69.5 (11.8)
**Body mass index**^**a**^ *(mean [sd])*	30.8 (6.1)
**Charlson Comorbidity Index**^**a,b**^ *(no. [%])*	
0	388 (56.4)
1 to 15	272 (39.5)
16 to 30	25 (3.6)
31 to 50	3 (0.4)
**Indication for Revision**^**a**^ *(no. [%])*	
Infection	345 (50.2)
Malalignment	10 (1.5)
Aseptic loosening/lysis	116 (16.9)
Component wear	14 (2.0)
Dislocation/instability	79 (11.5)
Periprosthetic fracture	19 (2.8)
Progressive arthritis	57 (8.3)
Stiffness	17 (2.5)
Unexplained pain	28 (4.1)
Other	3 (0.4)

^a^Data aggregated on a procedure level from the perspective of the surgical unit

^b^Summary Hospital-level Mortality Indicator specification

### Indication for surgery

There were 170 (24.7%) unplanned admissions and 518 (75.3%) elective admissions for revision TKR. The indication for rTKR was: infection (*n* = 345, 50.2%); aseptic loosening/lysis (*n* = 116, 16.9%); dislocation/instability (*n* = 79, 11.5%); progressive arthritis (*n* = 57, 8.3%); unexplained pain (*n* = 28, 4.1%); periprosthetic fracture (*n* = 19, 2.8%); stiffness (*n* = 17, 2.5%); component wear (*n* = 14, 2.0%); malalignment (*n* = 10, 1.5%); and other (*n* = 3, 0.4%). The revision diagnosis is plotted against the number of revision procedures in Fig. 1b.

### Referral pattern

**Fig. 2:**
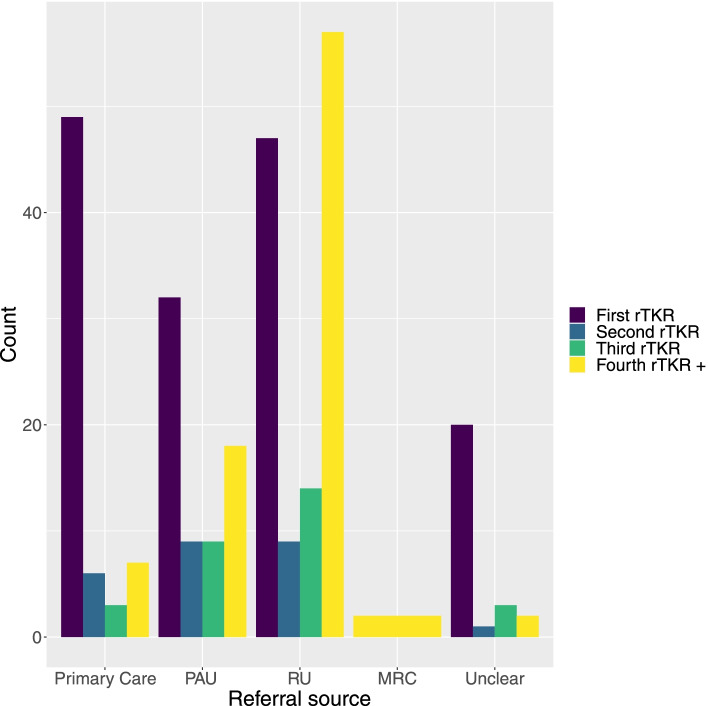
Bar-charts demonstrating referral sources to the major revision centre for first and subsequent revision procedures. 140/288 (48.6%) of patients were referred to the MRC after their first revision procedure. PAU Primary arthroplasty unit, RU Revision unit, MRC Major revision centre

Among the 534 patients who underwent rTKR over the study period, 288 (53.9%) patients were new referrals to the MRC, 209 (39.1%) patients had a previous primary knee replacement at the MRC and 37 (6.9%) patients had a previous rTKR at the MRC. Among the 288 new referrals, the referral source was: primary care (65 rTKR, 22.5%); primary arthroplasty unit (68 rTKR, 23.6%); revision unit (127 rTKR, 44.1%); major revision centre (2 rTKR, 0.7%); and not available (26 rTKR, 9.0%). The two quaternary referrals from other major revision centres were both multiply-revised patients presenting with prosthetic joint infection to our Bone Infection Unit. 140/288 (48.6%) of the referred cases had previously undergone one or more rTKR prior to referral. The pattern of referrals is illustrated in Fig. 2.

### Technical complexity of surgery

**Fig. 3:**
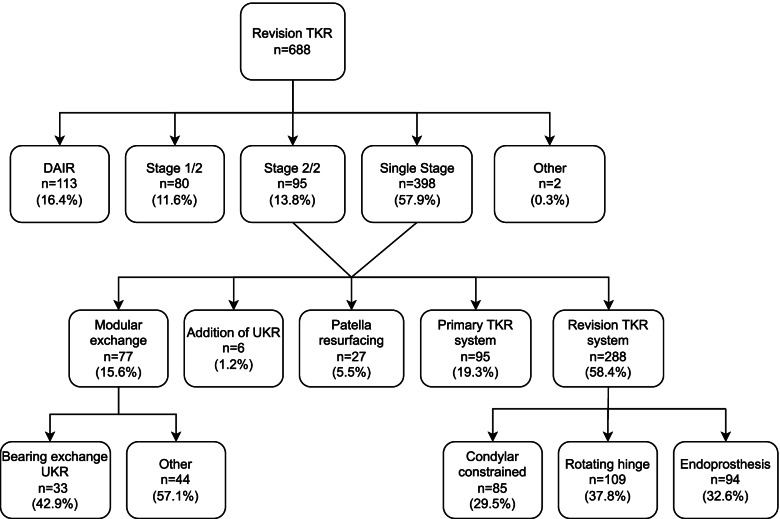
Flowchart demonstrating the technical details of revision TKR procedures performed over the study period. [Footnotes – DAIR debridement, antibiotics and implant retention, TKR total knee replacement, UKR unicompartmental knee replacement]

The overall complexity of the 688 rTKR procedures performed over the study period was: R1 *n* = 295 knees (42.9%), R2 *n* = 181 knees (26.3%) and R3 *n* = 212 knees (30.8%). The surgical complexity of the 288 knees referred from external units was: R1 *n* = 96 knees (33.3%), R2 *n* = 92 knees (31.9%) and R3 *n* = 100 knees (34.7%). Among the 209 knees that underwent rTKR after primary knee replacement at our centre, the surgical complexity was: R1 *n* = 138 (66.0%), R2 *n* = 38 (18.2%) and R3 *n* = 33 (15.8%). The technical details of revision procedures performed over the study period are described in Fig. 3.

### Hospital admission impact

**Fig. 4:**
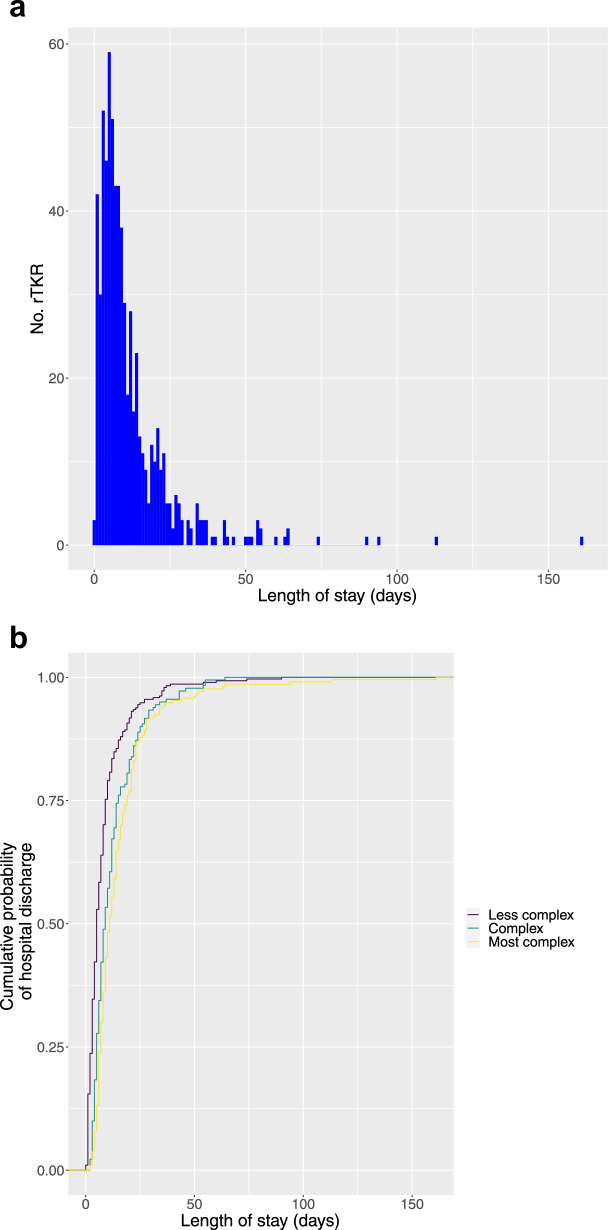
**a** Histogram demonstrating length of hospital stay following rTKR. **b** Cumulative probability of hospital discharge by length of stay and complexity of rTKR (rated according to RKCC as ‘less complex’, ‘complex’ or ‘most complex’)

A histogram of length of stay (LOS) is provided in Fig. 4a. This demonstrated a positive skew, with median LOS 8 days (interquartile range 4–14). Higher surgical complexity was associated to longer hospital stay (Spearman rank correlation coefficient 0.38, *p* < 0.0001). The relationship between RKCC and LOS is illustrated in Fig. 4b. ‘Less complex’ cases had median LOS 5 days (IQR 3 – 9); ‘complex’ cases had median LOS 9 days (IQR 5 – 15); and ‘most complex/salvage’ cases had median LOS 11 days (IQR 7 – 19).

Three hundred thirty out of 688 patients (48.0%) were treated in higher-dependency care for at least one night following rTKR. Most of these patients (*n* = 317) were observed overnight in an enhanced care environment within our theatre recovery suite on the day of surgery. However, 12 patients out of 688 (1.7%) required transfer out of our centre for formal admission to HDU (for 2 nights [*n* = 9], 3 nights [*n* = 2] and 5 nights [*n* = 1]). One patient out of 688 (0.1%) was transferred to the intensive care unit (ICU) due to an aspiration pneumonia perioperatively.

## Discussion

This study has described the surgical practice, patterns of referral and resource utilisation of revision TKR at a major revision centre. The surgical complexity of rTKR procedures performed at the MRC obeyed a ‘rule of thirds’: one-third of cases ‘less complex’, one-third ‘complex’ and one-third the ‘most complex/salvage’. Among external referrals, nearly half of patients had a previous rTKR prior to referral.

We found that higher case complexity was associated to longer length of hospital stay. The ‘most complex’ cases had a median LOS of 11 days, compared to 5 days for ‘less complex’ cases. Assessment of bed capacity will be essential prior to service reconfiguration to prevent ‘log-jams’ in the system. The approach to this problem in the context of Trauma networks involves ‘repatriation’ of patients to local units after surgery and similar arrangements may be needed for rTKR. Length of stay is also an important driver of hospital costs. Petrie et al. [18] recently showed that more complex cases were inadequately reimbursed in the NHS model of care by the national tariff, indicating the need for financial uplifts to incentivise this work. One significant financial burden and potential barrier to centres planning to deliver rTKR is the requirement for higher-dependency care. In our centre, we found that nearly half of all patients undergoing rTKR received (mostly planned) ‘enhanced’ care overnight on the day of surgery (which does not carry additional remuneration) with few patients (< 2%) requiring transfer out of our centre for formal HDU/ICU admission. This information is of current relevance given long waiting lists and calls for investment in ‘surgical hubs’ [19]. A further financial burden to consider for major revision centres is the need to maintain an extensive inventory of revision implant systems. These costs should be reflected in the remuneration of major revision centres – and are likely to be more cost-efficient for the healthcare system overall when compared with large numbers of units ‘loaning in’ kits.

The methods used in this study are suitable for implementation by other surgical units to gain a more detailed understanding of their own operative practices, which may be used for local service planning. Our approach is likely to provide an adjunct to models analysing routinely collected data [8] and may overcome some of the limitations associated with this. One important finding here was that publicly-reported rTKR activity by NHS Digital underestimated actual operative activity. For example, in 2016/7 the rTKR workload reported by HES was 35.6% lower than that identified from our local database. The source of this discrepancy requires further investigation as it may be relevant for hospital reimbursement and the interpretation of publicly reported surgical outcomes [20].

This study has a number of limitations that should be considered. First, the recruitment of cases from theatre logbooks means that only cases managed with surgery were enrolled. As such, the wider role of a major revision centre in providing multidisciplinary team advice, specialist diagnostics and non-operative interventions (with the associated financial implications and pressure on MRC resources) has not been measured. Second, the generalisability of our findings to other major revision centres has not been tested. The high observed prevalence of prosthetic joint infection among referrals is likely to reflect the presence of a specialist Bone Infection Unit at our centre and may be lower in other units. Our study was performed in the UK in the context of an NHS provider. As such our findings may not be generalisable to other countries or healthcare models. Our centre has a high utilisation of unicompartmental knee replacement, which may explain why we observed a high prevalence of ‘less complex’ rTKRs in patients who had their original knee replacement at our centre. A final limitation to consider is that, whilst the RKCC has previously been shown to have good intra- and inter-observer reliability, there may be subjectivity in the classification of some cases [12].

The question as to whether revision surgery in a network model will lead to better surgical outcomes has not been answered here. However, this principle is accepted for other sub-specialties, including major trauma and sarcoma. We have already introduced the idea above of cost-savings through economies of scale – for example, through reduction of “loan kits”. There is a need for high-quality research to investigate the association of surgeon and unit caseload with clinical outcomes following rTKR. In addition, the implementation of a network model must be sensitive to local intricacies in service provision and the process must involve the surgeons practising in these units as well as patient group representatives [21]. It is important to recognise the impact of centralisation on the patient experience. For example, centralisation may be associated with longer travel times for specialist opinions, surgery and follow-up appointments. Patients may have developed good rapport with their primary surgeon and – particularly for high-volume, highly-skilled primary arthroplasty surgeons with low-volume revision practices, but achieving good outcomes – it may be that some revisions would achieve a better patient experience for an equivalent outcome without tertiary referral.

In conclusion, the surgical complexity of rTKR procedures at our MRC was distributed evenly as ‘less complex’, ‘more complex’ and ‘the most complex’ cases. Higher surgical complexity was associated to greater length of hospital stay. Among external referrals, nearly half of patients had a previous rTKR prior to referral. This information is likely to benefit service planning for the development of revision knee arthroplasty networks in the near future.

## Supplementary Information

**Additional file1.** Appendix 1.NHS Digital OPCS Codes used tocalculate summary statistics for revision knee replacement

**Additional file2.** Appendix 2.Diagnosis hierarchy for revision total kneereplacement (based on AOANJRR) [3]

**Additional file3.** Appendix 3.Revision Knee Complexity Classification (RKCC) –adapted from Phillips et al [15]
