# Supplementary material for: Revision total knee replacement case-mix at a major revision centre
Source: J Exp Orthop. 2022 Apr 14;9:34. doi: 10.1186/s40634-022-00462-2 (PMC9010489; doi:10.1186/s40634-022-00462-2)
Supplement: Supplementary file 1 — Additional file1. Appendix 1.NHS Digital OPCS Codes used tocalculate summary statistics for revision knee replacement [file 40634_2022_462_MOESM1_ESM.docx]

**Appendix 1**

*NHS Digital OPCS Codes used to calculate summary statistics for revision knee replacement*

| *OPCS Code* | *Note(s)* | *Code Description* |
| --- | --- | --- |
| O180 |  | Conversion from previous hybrid prosthetic replacement of knee joint using cement |
| O182 |  | Conversion to hybrid prosthetic replacement of knee joint using cement |
| O183 |  | Revision of hybrid prosthetic replacement of knee joint using cement |
| O184 |  | Attention to hybrid prosthetic replacement of knee joint using cement |
| W400 |  | Conversion from previous cemented total prosthetic replacement of knee joint |
| W402 |  | Conversion to total prosthetic replacement of knee joint using cement |
| W403 |  | Revision of total prosthetic replacement of knee joint using cement |
| W404 |  | Revision of one component of total prosthetic replacement of knee joint using cement |
| W410 |  | Conversion from previous uncemented total prosthetic replacement of knee joint |
| W412 |  | Conversion to total prosthetic replacement of knee joint not using cement |
| W413 |  | Revision of total prosthetic replacement of knee joint not using cement |
| W414 |  | Revision of one component of total prosthetic replacement of knee joint not using cement |
| W420 |  | Conversion from previous total prosthetic replacement of knee joint NEC |
| W422 |  | Conversion to total prosthetic replacement of knee joint NEC |
| W423 |  | Revision of total prosthetic replacement of knee joint NEC |
| W424 | A | Attention to total prosthetic replacement of knee joint NEC |
| W425 |  | Revision of one component of total prosthetic replacement of knee joint NEC |
| W426 |  | Arthrolysis of total prosthetic replacement of knee joint |
| W520 | K | Conversion from previous cemented prosthetic replacement of articulation of bone NEC |
| W522 | K | Conversion to prosthetic replacement of articulation of bone using cement NEC |
| W523 | K | Revision of prosthetic replacement of articulation of bone using cement NEC |
| W530 | K | Conversion from previous uncemented prosthetic replacement of articulation of bone NEC |
| W532 | K | Conversion to prosthetic replacement of articulation of bone not using cement NEC |
| W533 | K | Revision of prosthetic replacement of articulation of bone not using cement NEC |
| W540 | K | Conversion from previous prosthetic replacement of articulation of bone NEC |
| W542 | K | Conversion to prosthetic replacement of articulation of bone NEC |
| W543 | K | Revision of prosthetic replacement of articulation of bone NEC |
| W544 | K, A | Attention to prosthetic replacement of articulation of bone NEC |
| W582 | K | Revision of resurfacing arthroplasty of joint |
|  |  |  |
| K | Must be combined with knee body part codes (Z846 Or Z765 Or Z845 or Z844 Or Z774 Or Z787) | |
| A | Must be combined with action codes (Y032 Or Y037) | |

*OPCS - Office of Population Censuses and Surveys Classification of Surgical Operations and Procedures (OPCS-4) codes used in Hospital Episode Statistics Admitted Patient Care*
