# Supplementary material for: Revision total knee replacement case-mix at a major revision centre
Source: J Exp Orthop. 2022 Apr 14;9:34. doi: 10.1186/s40634-022-00462-2 (PMC9010489; doi:10.1186/s40634-022-00462-2)
Supplement: Supplementary file 2 — Additional file2. Appendix 2.Diagnosis hierarchy for revision total kneereplacement (based on AOANJRR) [3] [file 40634_2022_462_MOESM2_ESM.docx]

**Appendix 2**

*Diagnosis hierarchy for revision total knee replacement (based on AOANJRR)* [3]

| **Rank** | **Diagnosis** |
| --- | --- |
| 1 | Infection |
| 2 | Malalignment |
| 3 | Aseptic loosening / lysis |
| 4 | Component wear |
| 5 | Dislocation / instability |
| 6 | Periprosthetic fracture |
| 7 | Progression of arthritis |
| 8 | Stiffness |
| 9 | Unexplained pain |
| 10 | Other |
