# Supplementary material for: Revision total knee replacement case-mix at a major revision centre
Source: J Exp Orthop. 2022 Apr 14;9:34. doi: 10.1186/s40634-022-00462-2 (PMC9010489; doi:10.1186/s40634-022-00462-2)
Supplement: Supplementary file 3 — Additional file3. Appendix 3.Revision Knee Complexity Classification (RKCC) –adapted from Phillips et al [15] [file 40634_2022_462_MOESM3_ESM.docx]

**Appendix 3**

*Revision Knee Complexity Classification (RKCC) – adapted from Phillips et al* [15]

***R1 (Revision 1)—Less complex revision surgery***

For example:

- First revision TKR for aseptic loosening
- Revision of a partial knee replacement to a total knee replacement
- Polyethylene exchange
- Debridement, antibiotics and implant retention (DAIR) procedures
- No additional complexity factors (such as patient comorbidities or soft tissue inadequacy)

***R2 (Revision 2)—Complex revision surgery***

For example:

- First re-revision operation
- First revison for PJI
- Bone loss requiring supplemental fixation (e.g. using a cone or sleeve) (AORI 2B)
- R1 cases with additional complexity factors

***R3 (Revision 3)—Most complex and salvage cases***

For example:

- Multiple previous revision procedures
- Bone loss requiring extensive metaphyseal reconstruction or massive endoprosthesis
- Requirement for hinged prosthesis due to bone loss or instability
- Salvage procedures (e.g. arthrodesis, amputation)
